# The Whisky Judgment

**Published:** 1906-03-03

**Authors:** 


### The Hospital.

A Journal of

The Medical Sciences and Hospital Administration.

Vol. XXXIX.—No. 1,014. SATURDAY, MARCH 3, 1906.

### THE WHISKY JUDGMENT.

Whatever may be the value to our readers of the technical information elicited during the protracted whisky inquiry at Islington, there can be no doubt of the interest to medical men of Mr. Fordham's judgment on Monday. We cannot in this short leader place our readers *au courant* with the subject of whisky in all its aspects, but we hope in an early issue to place before them an account of the history, manufacture, sale, and nature of whisky, based upon an inquiry instituted by ourselves. Meanwhile, it will be well to consider what the Islington magistrate's decision is, and what it means. If we crystallize out the meaning from Mr. Fordham's 5,000 words, we find that to be whisky a spirit must be distilled in a particular form of still—namely, the pot-still, and from a mash consisting in Scotland of malted barley, and in Ireland of 75 per cent. of malted barley and 25 per cent. of *indigenous* grain.

This, indeed, is a definition of mathematical precision, and who runs may read. Unfortunately the subject of whisky has a literature, and the magisterial mind has rushed in where select committees and the legislature have feared to tread. In a word, Mr. Fordham's judgment completely reverses the finding of a Select Committee of the House of Commons on British and Foreign Spirits, which sat for two years, and reported in 1891; also, in principle, it is diametrically opposed both to the report of the Beer Materials Committee of 1898, and to the spirit of the legislation on the subject as expressed by the Free Mash Tun Act of 1881. The subject matter considered by the Select Committee on British and Foreign Spirits was identical with that placed before Mr. Fordham. It seems *a priori* extraordinary that a police magistrate should accomplish the *tour de force* of not only reversing, but also of entirely ignoring the labours of a Select Committee comprising among its members Lord Playfair and Sir Henry Roscoe; and amongst its witnesses the most eminent medical and chemical experts of the day. But much more remarkable is the fact that if Mr. Fordham's judgment is correct, distillers, technologists, and members of the spirit trade, are ruthlessly informed, that what they and their grandfathers have sold as whisky is not whisky at all, and medical men and the public are equally ruthlessly told that what they have prescribed, drunk, and enjoyed as whisky for the last fifty years, is neither Scotch nor Irish whisky.

In our opinion such a *bouleversement* of the established order of things could only be justified by very cogent reasons. We have examined Mr. Fordham's voluminous judgment carefully, and regret to find that such reasons are conspicuous by their absence.

Mr. Fordham's objections to nine-tenths of the spirit consumed as whisky at the present time are threefold. He objects to the apparatus, to the materials, and to the properties of the finished product. Up to about 70 years ago (at which period the patent-still was introduced) whisky was undoubtedly exclusively made in a pot-still; therefore, says Mr. Fordham in effect, to be whisky it must be made in a pot-still at the present day. We believe that locomotion on the surface of water at the time of Noah was exclusively accomplished in an ark. Nowadays, nevertheless, some few of us venture to go down to the sea in ships propelled by steam-power. The thesis that an article of food or drink must always be prepared by the sole means of the apparatus originally used would absolutely stop any advancement in hygienic technology. As regards materials, Mr. Fordham objects to the use of maize. We would simply in this connection point out that the use of maize in brewing was legalised by the passing of a special Act, but that long previous to this Act the use of any cereal grain including maize was legal in the manufacture of whisky, and that the American Pharmacopœia, in which whisky is official, specifically states that it can be made from maize.

Mr. Fordham says that a finished product, largely composed of patent-still whisky, is entirely different in its properties from a pure pot-still whisky. Nobody denies that the flavour of these two products is different; in fact, it is precisely this difference of flavour which has led nine-tenths of the whisky-drinkers of to-day to prefer the former product. We are glad to find that even at Islington the fiction which has been propagated no doubt for interested trade reasons, that the product of the patent-still is deleterious to health, has exploded. In the face of Professor Tunnicliffe's evidence at

Islington and the evidence of Sir Lauder Brunton and others before the Select Committee, it would be odd if this were not so. We have little doubt that medical men are now well aware of the fact that a blended whisky—that is, a whisky containing both patent-still and pot-still spirit, is not only preferred by their patients, but is probably better for them than a pure pot-still whisky.

Over and above the important but individual considerations involved in this case, a general defect in our legislative system is once more by it plainly discernible. It has for a long time been perfectly clear that a police court is not the proper tribunal for cases involving admittedly points of great technological and scientific complications. Several Select Committees have recommended a special court of reference, not only for deciding cases of this kind, but also for relieving the public analyst of responsibilities which sit ill upon his shoulders, and for the bearing of which he is inadequately qualified.

